# Hypervirulent K. Pneumoniae Secretes More and More Active Iron-Acquisition Molecules than “Classical” K. Pneumoniae Thereby Enhancing its Virulence

**DOI:** 10.1371/journal.pone.0026734

**Published:** 2011-10-24

**Authors:** Thomas A. Russo, Alyssa S. Shon, Janet M. Beanan, Ruth Olson, Ulrike MacDonald, Alexander O. Pomakov, Mark P. Visitacion

**Affiliations:** 1 Veterans Administration Western New York Healthcare System, University at Buffalo-State University of New York, Buffalo, New York, United States of America; 2 Department of Medicine, University at Buffalo-State University of New York, Buffalo, New York, United States of America; 3 Department of Microbiology and Immunology, University at Buffalo-State University of New York, Buffalo, New York, United States of America; 4 The Witebsky Center for Microbial Pathogenesis, University at Buffalo-State University of New York, Buffalo, New York, United States of America; Los Angeles Biomedical Research Institute, United States of America

## Abstract

**Background:**

A new hypervirulent (hypermucoviscous) clinical variant of *Klebsiella pneumoniae* (hvKP) has emerged over the last decade. Our goal is to identify new mechanisms, which increase the virulence hvKP compared to “classic” *K. pneumoniae* (cKP).

**Methodology/Principal Findings:**

Various growth assays were performed in human ascites, human serum, and laboratory medium with the hvKP strain hvKP1 (wt), randomly chosen blood isolates of cKP strains (cKP1-4), and mutant constructs deficient in the secretion of selected compounds. An in vivo mouse model that mimics infection due to hvKP and a quantitative siderophore assay were also used. It was established that a molecule(s)/factor(s) was secreted by hvKP1 significantly enhanced its growth and/or survival in human ascites. This molecule(s)/factor(s) also increased the growth and/or survival of hvKP1 in serum ex vivo and in an in vivo mouse model that measures metastatic spread after subcutaneous challenge, thereby further establishing biologic significance. Although features such as a size of <3kD, heat stability, and growth characteristics in ascites suggested this molecule(s) was a quorum-sensing compound, data presented demonstrates that this molecule(s)/factor(s) is involved in iron uptake and is likely a siderophore(s) or another iron-acquisition molecule. Although it is known that iron acquisition is critical for virulence, a novel aspect of this observation is that hvKP1 produces quantitatively more siderophores that appear to be biologically more active (increased affinity for iron or more resistant to host factors) than those produced by cKP strains.

**Conclusions/Significance:**

The data presented delineates a new mechanism by which hvKP increases its pathogenic potential compared to cKP strains. This paradigm may be broadly applicable to other extraintestinal gram-negative bacilli.

## Introduction

Until recently, most *Klebsiella* infections were due to “classic” *K. pneumoniae* (cKP) strains. Particularly in developed Western countries most infections occurred in hospitals and long-term care facilities. Pneumonia, urinary tract infection, abdominal infection, intra-vascular device infection, surgical site infection, soft tissue infection, and subsequent bacteremia were the most common clinical syndromes.

However a new, hypervirulent clinical variant of *Klebsiella pneumoniae* (hvKP) has emerged over the last decade. Initial reports were from the “Pacific Rim”, but more recently hvKP has been an emerging pathogen in the United States, Canada, Europe, Israel, South Africa, Australia and elsewhere [1], [2], [3], [4], [5]. In fact, a case of hvKP infection in Buffalo, New York, USA was one of the motivating factors for our laboratory to study hvKP [6]. At first, infection due to hvKP was clinically defined and distinguished from traditional infections due to cKP by: 1) presenting as community-acquired liver abscess (CA-PLA), 2) affecting patients lacking a history of hepatobiliary disease, and 3) a propensity for causing metastatic spread to distant sites in 11-80% of cases (e.g. lungs, pleura, prostate, bone, joints, kidneys, spleen, muscle/fascia, soft-tissue, skin, eyes, and central nervous system (CNS) [2], [4], [7], [8], [9], [10], [11], [12], [13]. hvKP infection frequently occurs in diabetics and Asians, but hosts are often young and healthy and infections are described in all ethic groups. hvKP is associated with a significant mortality rate, ranging from 3–32% [7], [9], [10], [14], [15], [16]. Further, survivors with metastatic spread often suffer catastrophic morbidity such as loss of vision and neurologic sequelae [7], [10]. From a clinical perspective, hvKP possesses novel features for an enteric gram-negative bacillus (GNB). Invasive infection (e.g. liver abscess) is an unusual occurrence for a healthy individual. Further, although metastatic spread is common for certain gram-positive pathogens such as *Staphylococcus aureus* and *Streptoccocci*, it is uncommon for extraintestinal GNB such as Klebsiella.

The lack of an unequivocal genotypic/phenotypic marker(s) for hvKP has precluded a comprehensive understanding of the prevalence and spectrum of hvKP disease. Although there is no clear surrogate marker for hvKP strains, a hypermucoviscous phenotype has been associated with strains that cause CA-PLA. This phenotype has been semi-quantitatively defined by a positive “string test” (formation of a viscous string >5mm in length when bacterial colonies on an agar plate are stretched by an inoculation loop). However, if a hypermucoviscous phenotype is a valid marker, the spectrum and number of infections due to hvKP is more extensive than appreciated. A variety of community-acquired, primary, non-hepatic infections including renal, deep neck, and parotid gland abscesses; mycotic aneurysm, osteomyelitis, subdural empyema, pulmonary empyema, and septic arthritis without concomitant hepatic abscess have been described [14], [17]. In a series of 200 cases of community acquired KP bacteremia, 42% (83/200) possessed a hypermucoviscous phenotype. Of these 83 cases, 23% were primary bacteremias, 21% were primary pneumonias, 11% were UTIs, 2% were spontaneous bacterial peritonitis, and 37% were the pathognomic CA-PLA ± metastatic spread to the CNS, eye, or pleural space [18]. The 14-day mortality in these 83 patients was 27%. A number of adult cases of primary community-acquired meningitis due to *K. pneumonia* have been reported from Taiwan, with mortality rates ranging from 30–83% and survivors suffering significant neurologic sequelae [16], [19], [20], [21]. Likewise, *K. pneumonia* was the second most common pathogen responsible for splenic abscess in a recent series from Korea [22]. The presence or absence of a hypermucoviscous phenotype was not reported in these studies, but since KP is an exceedingly rare cause of primary meningitis and splenic abscess in adults, it is tempting to speculate that hvKP was the responsible pathogen.

Compounding an already challenging clinical situation is the recent propensity of *K. pneumoniae* to become multi-dug resistant (MDR), including the acquisition of extended-spectrum β-lactamases and carbapenemases, such as the recently described New Delhi metallo-β-lactamase (NDM-1) [23]. Some cases of infection due to hvKP caused by MDR-strains have already been described [24] and as expected, outcome is worse with inappropriate treatment [25]. As a result, management of infections due to hvKP will become extremely challenging and morbidity and mortality rates will increase. The confluence of hypervirulence and MDR in hvKP has the potential to create a “post-antibiotic” scenario; similar to what was feared with methicillin resistant *S. aureus* (MRSA) but was never realized. Therefore, enhancing our understanding of this highly virulent pathogen is critical. In this report we describe a novel mechanism that increases the virulence of hvKP and discuss these new finding within the context of what is known about the virulence of hvKP.

## Results

### hvKP1, when sub-cultured from LB medium, is able to grow in human ascites at a starting inoculum of 10^7^ cfu/ml, but not at 10^3^ and 10^5^ cfu/ml

**Figure 1:**
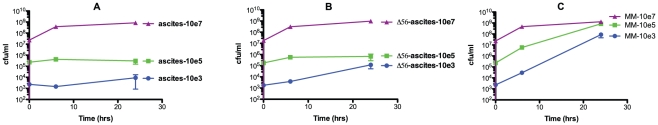
Growth and/or survival of hvKP1 (wt) in 100% human ascites and M9 minimal medium. The growth and/or survival of hvKP1 was assessed at 0, 3, 6, and 24 hours in 100% human ascites (batch 2) or M9 minimal medium at the starting inocula of approximately 10^3^ cfu/ml, 10^5^ cfu/ml, and 10^7^ cfu/ml. Panel A: 100% human ascites, Panel B: 100% human ascites heated at 56°C for 30 minutes, and Panel C: M9 minimal medium. Data are Mean ± S.E.M.; N = 3.

Human ascites is a body fluid that mimics the environment within the human host in which extracellular pathogens such as Klebsiella grow and survive. The growth and/or survival of hvKP1 was characterized in ascites (batch 2) after overnight growth in LB medium. Somewhat surprisingly, growth was limited when the starting inoculum was 10^3^ cfu/ml and 10^5^ cfu/ml, but not 10^7^ cfu/ml (Figure 1A). It was then hypothesized that this growth difference may be due to the bactericidal effects of complement, which is present in ascites. So next, the growth and/or survival of hvKP1 (sub-cultured from LB medium) was characterized in the same batch of ascites that had been heated at 56°C for 30 minutes (which inactivated the bactericidal activity of complement). However, the growth and/or survival of hvKP1 was again limited when the starting inoculum was 10^3^ cfu/ml and 10^5^ cfu/ml, but not 10^7^ cfu/ml (Figure 1B). By contrast, hvKP1 was able to grow from starting inocula of 10^3^, 10^5^, and 10^7^ cfu/ml in minimal medium, achieving a plateau density of 8.8×10^7^, 8.5×10^8^ and 1.9×10^9^ cfu/ml respectively (Figure 1C). These data lead to the hypothesis that quorum sensing or a quorum sensing-like mechanism was responsible for the observed growth patterns in human ascites.

### The addition of conditioned medium generated by hvKP1 grown in human ascites and minimal medium, but not LB broth, facilitates growth of hvKP1 in human ascites at a starting inoculum of 10^3^ cfu/ml

**Figure 2:**
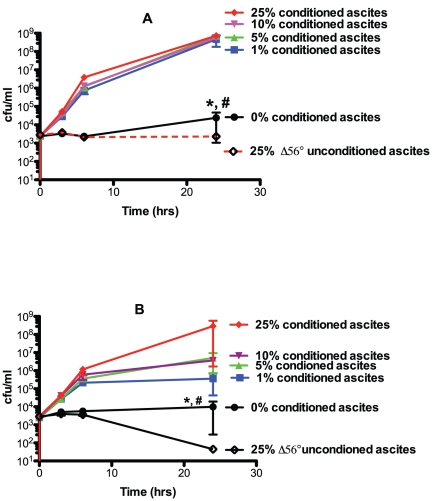
Growth and/or survival of hvKP1 grown in human ascites supplemented with varying concentration of homologous, conditioned ascites. The growth and/or survival of hvKP1 was assessed at 0, 3, 6, and 24 hours in 100% human ascites supplemented with homologous conditioned ascites resulting in final concentrations of 0%, 1%, 5%, 10%, and 25%. Growth and/or survival was also assessed in ascites supplemented with 25% unconditioned ascites heated at 56°C for 30 minutes to control for a possible effect of conditioned ascites diluting complement-mediated bactericidal activity. Panel A: ascites batch 1. Panel B: ascites batch 2. Supplementation with 5%, 10%, and 25% conditioned, homologous ascites, compared to 0%, resulted in a significant increase in growth and/or survival for both batches of ascites (* P<0.05/4) and supplementation with 1% conditioned, homologous ascites resulted in a trend for increased growth and/or survival (# P>0.05/4 and<0.05). Data are Mean ± S.E.M.; N = 2–3.

Quorum sensing is a cell-to-cell signaling mechanism that facilitates communal growth via secreted molecules termed autoinducers (AI) [26], [27], [28]. A high density of organisms is often requisite for the concentration of the secreted AI to achieve the necessary concentration to regulate gene expression. Such concentrations are often achieved in late logarithmic-to-stationary phase grown cells. Therefore, we generated bacterial-free supernatant from stationary phase hvKP1 grown in human ascites, minimal medium, and LB broth (conditioned media) to test its effects on the growth of hvKP1 in human ascites at a starting inoculum of approximately 10^3^ cfu/ml. When grown in two independent batches (batches 1 and 2) of human ascites with final concentrations of 5%, 10%, and 25% homologous conditioned ascites, a significant increase in the growth and/or survival of hvKP1 was observed compared to unconditioned ascites and a trend was observed with 1% supplementation (Figure 2). By contrast, no growth was observed with final concentrations of 0% conditioned ascites and 25% of unconditioned ascites heated to 56°C for 30 minutes. This later condition served as a control for the addition of conditioned ascites diluting complement-mediated bactericidal activity in ascites. These data support the hypothesis that a factor is present in conditioned ascites that promotes the growth and/or survival of a low starting inoculum of hvKP1 in human ascites.

**Figure 3:**
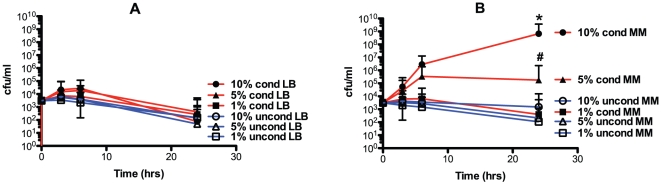
Growth/survival of hvKP1 grown in human ascites supplemented with hvKP1 generated M9 and LB conditioned media. The growth of hvKP1 was assessed at 0, 3, 6, and 24 hours in human ascites (batch 2) supplemented with unconditioned (uncond) or hvKP1 generated conditioned (cond) rich laboratory (LB) or minimal (M9) media resulting in final concentrations of 0%, 1%, 5%, and 10%. Panel A: ascites supplemented with conditioned rich laboratory medium (LB). Panel B: ascites supplemented with conditioned minimal medium (MM). Supplementation with 10% conditioned MM compared to 0% resulted in a significant increase in growth/survival (* P<0.05/3) and supplementation with 5% conditioned MM compared to 0% resulted in a trend towards an increase in growth/survival (# P>0.05/3 and<0.05). Data are Mean ± S.E.M.; N = 2.

Next, the effect of LB (iron replete) and M9 (partially iron depleted) conditioned media (generated by growth of hvKP1 in these media overnight) on the growth and/or survival of hvKP1 in human ascites (batch 2) at a starting inoculum of approximately 10^3^ cfu/ml was assessed. Growth and/or survival was not affected when hvKP1 was grown in human ascites with final concentrations of 1%, 5%, and 10% conditioned LB medium or unconditioned media. By contrast, a significant increase in the growth and/or survival of hvKP1 was observed when grown in human ascites with final concentrations of 10% conditioned minimal medium, a trend towards increased growth/survival was observed with final concentrations of 5% conditioned minimal medium, but not 1% of conditioned minimal medium compared to the respective concentrations of unconditioned minimal medium (Figure 3). However, a higher final concentration of conditioned minimal medium was necessary, compared to conditioned ascites. These data support the hypothesis that a factor is present in conditioned minimal medium, but not conditioned LB medium, that promotes the growth and/or survival of a low starting inoculum of hvKP1 in human ascites and that this factor may be iron-regulated. These data also demonstrate that the factor is derived from hvKP1 since there are no host factors present in minimal medium.

### The presence conditioned ascites or minimal medium increases the growth and/or survival of hvKP1 in 80% human serum

**Figure 4:**
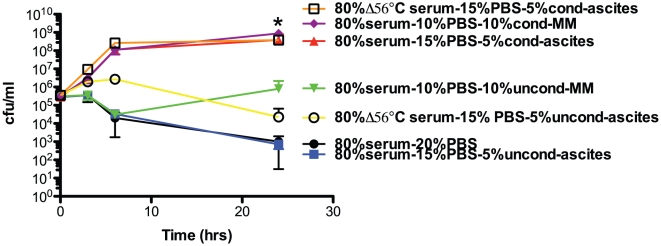
The survival of hvKP1 in 80% human serum is significantly increased when serum is supplemented with hvKP1 generated conditioned ascites or minimal medium. The survival of hvKP1 in 80% active or heat inactivated (56°C for 30 minutes (Δ56°C)) human serum was assessed at 0, 3, 6, and 24 hours. Growth in heat inactivated serum served was a positive control that established that decreased survival was due to complement-mediated bactericidal activity. The active serum was supplemented with final concentrations of: 1) 20% 1×phosphate-buffered saline (PBS), 2) 15% PBS plus 5% unconditioned ascites (uncond-ascites; batch 2), 3) 10% PBS and 10% unconditioned M9 minimal medium (uncond-MM), 4) 15% PBS and 5% conditioned ascites (cond-ascites; batch 2), and 5) 10% PBS and 10% conditioned M9 minimal medium (cond-MM). The heat inactivated serum was supplemented with final concentrations of: 1) 15% PBS and 5% unconditioned ascites (uncond-ascites) and 2) 15% PBS and 5% conditioned ascites (cond-ascites). Survival of hvKP1 was significantly increased when 80% active serum was supplemented with hvKP1-conditioned ascites compared to unconditioned ascites (*P<0.05/1) or when supplemented with conditioned M9 minimal medium compared to unconditioned M9 minimal medium (*P<0.05/1). Survival of hvKP1 was significantly increased when 80% heat inactivated serum was supplemented with hvKP1-conditioned ascites compared to unconditioned ascites (*P<0.05/1). Data are Mean ± S.E.M.; N = 3–4.

Human ascites contains a variable amount of complement. The ability of conditioned ascites and minimal medium to increase growth and/or survival of hvKP1 in this clinically relevant body fluid is biologically significant. To extend this observation, the effect of conditioned ascites and minimal medium was assessed in 80% human serum, a more quantitatively defined and challenging growth environment for hvKP1. A starting inoculum of approximately 1×10^5^ cfu/ml was chosen since hvKP1 did not grow in ascites at this titer and our group for serum sensitivity experiments has used this inoculum historically [6], [29], [30]. The growth and/or survival of hvKP1 when grown in 80% serum plus final concentrations of either 5% conditioned ascites (batch 2) or 10% conditioned minimal medium was significantly greater than in 80% serum plus either 5% unconditioned ascites or 10% unconditioned minimal medium respectively (Figure 4). These data further support the biologic significance of the effect of conditioned ascites or minimal medium on the growth and/or survival of hvKP1 in the clinically relevant human body fluid.

### hvKP1 grown in conditioned ascites, compared to unconditioned ascites, were more virulent in a mouse systemic infection model

**Figure 5:**
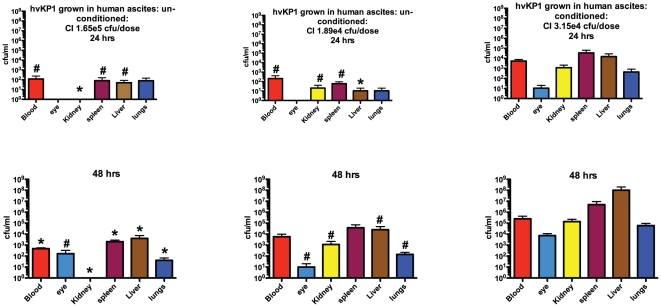
hvKP1 grown in conditioned ascites, compared to unconditioned ascites, resulted in significantly greater growth and/or survival and dissemination after SQ challenge of CD1 mice. Animals were challenged subcutaneously with 3.15×10^4^ cfu of hvKP1 grown in conditioned ascites (batch 2) and 1.89×10^4^ cfu or 1.65×10^5^ cfu of hvKP1 grown in unconditioned ascites (batch 2)(n = 6 for each condition and challenge inocula). Bacterial titers were enumerated in blood and various organs at 24 and 48 hours after challenge. After blood was obtained for culture, the vasculture was “flushed” with 1xPBS and organs were subsequently harvested. Comparisons were between hvKP1 grown in conditioned ascites and hvKP1 grown in unconditioned ascites. * P<0.05/2; # P>0.05/2 but <0.1. Data are Mean ± S.E.M. for n =  3.

As the next step in establishing biologic relevance of the effect of growing hvKP1 in conditioned compared to unconditioned ascites, a mouse systemic infection model developed by our group was used. This model mimics metastatic spread of hvKP, which occurs in human infection. In addition, mice are challenged via a physiologically relevant route that avoids the confounding effect of a potential cytokine “storm” (see Methods for additional details). Outbred CD1 mice were challenged subcutaneously with 3.15×10^4^ cfu of hvKP1 grown in human ascites (batch 2) with a final concentration of 5% homologous conditioned ascites or with 1.89×10^4^ cfu or 1.65×10^5^ of hvKP1 grown in unconditioned human ascites (batch 2). The metastatic spread of hvKP1 from the subcutaneous compartment to the blood, eye, kidney, spleen, liver and lungs was greater when hvKP1 was grown in the presence of conditioned ascites, compared to unconditioned ascites at all times post-bacterial challenge (24 and 48 hours) and this difference was statistically significant or demonstrated a trend towards significance for most comparisons (Figure 5). These data further establish the ability of conditioned ascites to enhance the virulence of hvKP1 in a clinically relevant in vivo model.

### The AI-2 and AI-3 quorum-sensing molecules do not appear to be the mediators in hvKP1 generated conditioned ascites and minimal medium that enhance the growth and/or survival of hvKP1

**Figure 6:**
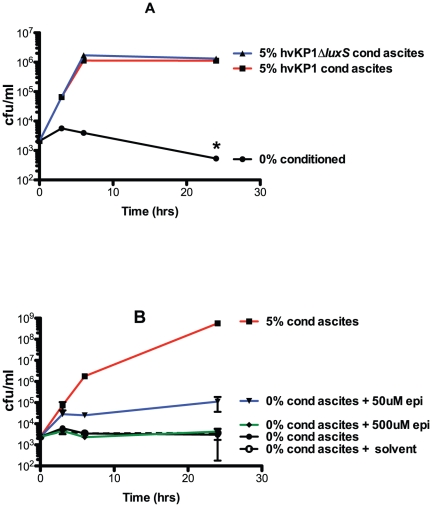
Neither LuxS generated AI-2 signaling molecules or the AI-3 signaling system appeared to affect the growth and/or survival of hvKP1 in conditioned ascites. The growth of hvKP1 was assessed at 0, 3, 6, and 24 hours in human ascites (batch 2). Panel A: Ascites was not supplemented (unconditioned) or supplemented with hvKP1 generated conditioned ascites (final concentration 5%) or hvKP1Δ*luxS* generated conditioned ascites (batch 2) (final concentration 5%). There was no significant difference in the growth of hvKP1 when supplemented with either of these conditioned media. Panel B: Ascites was not supplemented (unconditioned) or supplemented with hvKP1 generated conditioned ascites (batch 2) (final concentration 5%) or epinephrine (final concentrations 50 µM and 500 µM). There was no significant difference in the growth of hvKP1 with the addition of either of these supplements. Data are Mean ± S.E.M.; N = 2–3.

The next important question to resolve was the nature of the compound present in conditioned ascites or minimal medium that enhanced the growth and/or survival of hvKP1 in human ascites and serum ex vivo and in a mouse infection model. Data from initial growth experiments (Figure 1 A, B) suggested the compound was an AI molecule that mediated a quorum sensing response. To date, AI signaling molecules in GNB have been categorized into AI-1, AI-2, and AI-3 [27]. LuxS is an enzyme, which has been shown to generate AI-2 in Vibrio species [26], however, it also serves as a key enzyme in the activated methyl cycle which generates the methyl donor S-adenosyl-L-methione [28]. The role of AI-2 mediating quorum sensing in non-Vibrio genera has recently been questioned [27], [28], [31], [32]. However since *luxS* is present in Klebsiella and has been implicated in biofilm formation [33], [34], we assessed whether LuxS generated AI-2 signaling molecules may be responsible for the observed effects of conditioned ascites and minimal medium described above. A *luxS* gene disruption was generated in hvKP1 (hvKP1Δ*luxS*). Conditioned ascites (batch 2) was generated by overnight growth of hvKP1 and hvKP1Δ*luxS*. However, the growth and/or survival of hvKP1 when inoculated at low titer into human ascites (batch 2) was similarly increased with a final concentration of 5% conditioned ascites medium regardless of whether hvKP1 or hvKP1::*luxS* was used for its generation (Figure 6A). Therefore, these data did not implicate an AI-2 quorum-sensing system as a mediator of conditioned ascites-mediated increased growth and/or survival.

Next, we assessed whether an AI-3 signaling system may be responsible. Both epinephrine (at 50nM) and norepinephrine have been shown to serve as signaling molecules for AI-3 quorum-sensing system [31], [35]. Therefore, we assessed the effect of epinephrine on the growth and/or survival of hvKP1 when inoculated at low titer into human ascites (batch 2). No significant effect was observed at the 50nM, a concentration previously used successfully, nor at 500nM (Figure 6B). Therefore, these data did not implicate the AI-3 quorum-sensing system as a mediator of conditioned ascites-mediated increased growth and/or survival.

### The mediators present in conditioned minimal medium and ascites that enhance the growth and/or survival of hvKP1 are <3kD and heat stable

**Figure 7:**
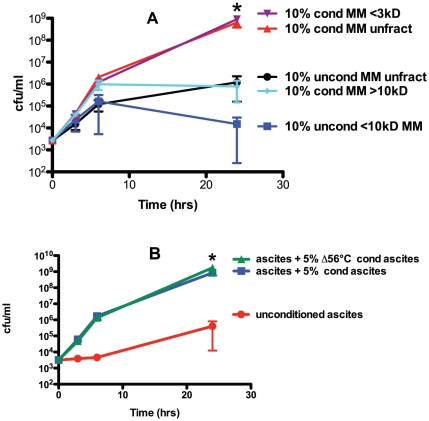
The mediators present in hvKP1 generated conditioned minimal medium and ascites that enhance the growth and/or survival of hvKP1 are <3kD and heat stable. The growth of hvKP1 was assessed at 0, 3, 6, and 24 hours in human ascites (batch 2). Panel A: Ascites was supplemented with either hvKP1 generated conditioned (cond) M9 minimal medium (MM) (10% final concentration) that was: 1) non-fractionated (unfract), 2) <3kD fraction, and 3) >10kD fraction or unconditioned (uncond) minimal medium (10% final concentration) that was: 4) non-fractionated, and 5) <10kD fraction. The growth and/or survival of hvKP1 was significantly increased when the ascites was supplemented with 10% conditioned, non-fractionated minimal medium or with 10% conditioned, <3kD fraction of minimal medium compared to 10% unconditioned, non-fractionated minimal medium and 10% unconditioned, <10kD fraction of minimal medium respectively (* P<0.5/1). Data are Mean ± S.E.M.; N = 2-3. Panel B: Ascites was not supplemented (unconditioned) or supplemented with hvKP1 generated conditioned (cond) ascites or with hvKP1 generated conditioned ascites that was heated at 56°C for 30 minutes (Δ56°C) (batch 2) (5% final concentration for both supplements). The growth and/or survival of hvKP1 was significantly increased when ascites was supplemented with either conditioned or conditioned medium Δ56°C compared to unconditioned ascites (* P<0.05/2). Data are Mean ± S.E.M.; N = 4.

To gain insight on the nature of the growth and/or survival-enhancing factor present in conditioned human ascites and minimal medium, size fractioning of the conditioned medium was performed. Conditioned minimal medium was used because size fractioning proved technically more challenging when ascites was used; presumably due to the host proteins present. The effect of a final concentration of: non-fractionated 10% conditioned or unconditioned minimal medium, 10% fractionated conditioned minimal media containing components <3kD or >10kD, and 10% fractionated unconditioned minimal media containing components <10kD on the growth and/or survival of hvKP1 in human ascites (batch 2) was assessed. The growth of hvKP1 was significantly greater when grown in ascites supplemented with non-fractionated conditioned minimal medium and fractionated conditioned minimal medium containing components <3kD compared to when grown in ascites supplemented with non-fractionated unconditioned minimal medium and fractionated unconditioned minimal medium containing components <10kD respectively. Therefore, the molecule or factor that mediated the growth and/or survival-enhancing effect was present in the fractionated conditioned medium containing components <3kD (Figure 7A).

Next, the stability of the factor or molecule after heating to 56°C for 30 minutes (Δ56°C) was assessed. The growth and/or survival of hvKP1 was significantly increased when ascites was supplemented with either 5% conditioned or 5% conditioned medium Δ56°C compared to unconditioned ascites (batch 2) (Figure 7B). Therefore the described heat treatment did not affect the activity of the molecule or factor in hvKP1-generated conditioned ascites (Figure 7B).

### An iron acquisition molecule(s) appears to be the mediator in hvKP1 generated conditioned ascites and minimal medium that enhances the growth and/or survival of hvKP1

**Figure 8:**
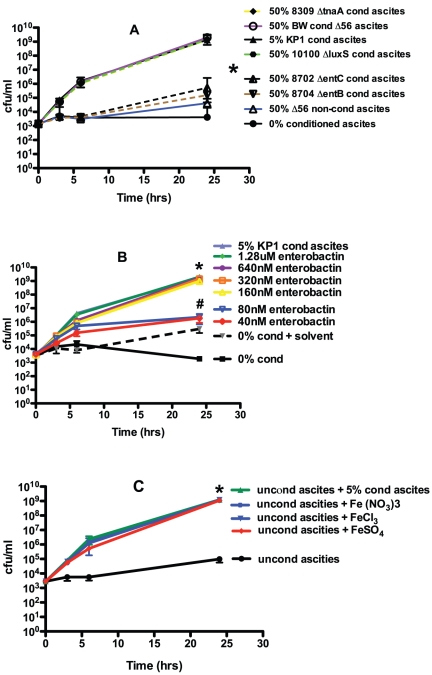
Identification of the class of mediators present in the hvKP1 generated conditioned minimal medium and ascites that enhance the growth and/or survival of hvKP1. The growth of hvKP1 was assessed at 0, 3, 6, and 24 hours in human ascites (batch 1). Panel A: Ascites was not supplemented (unconditioned) or supplemented with: 1) 5% hvKP1-generated conditioned (cond) ascites (batch 1) or 2) 50% conditioned ascites generated from the *E. coli* laboratory strain BW25113 and isogenic derivatives deficient in the production of enterobactin (BW25113Δ*entB* (CGSC#8704), BW25113Δ*entC* (CGSC#8702)), AI-2 molecules (BW25113Δ*luxS* (CGSC#10100)), and indole (BW25113Δ*tnaA* (CGSC#8309)) (batch 1). The growth and/or survival of hvKP1 was significantly decreased when grown in ascites supplemented with conditioned ascites generated by the *E. coli* strains deficient in enterobactin production compared to when grown in ascites supplemented with conditioned ascites generated by the *E. coli* strains sufficient in enterobactin production or with conditioned ascites generated by hvKP1 (*P<0.05/4). Data are Mean ± S.E.M.; N = 3. Panel B: Ascites (batch 1) was not supplemented (unconditioned) or supplemented with: 1) solvent alone (0.86% DMSO), 2) 5% hvKP1-generated conditioned (cond) ascites (batch 1), or 3) 40nM, 80nM, 160nM, 320nM, 640nM, and 1.28 µM of purified enterobactin. The growth and/or survival of hvKP1 was significantly increased when grown in ascites supplemented with 160nM, 320nM, 640nM, and 1.28 µM of purified enterobactin (*P<0.05/6) and there was a trend for an increase with supplementation with 40nM and 80nM of purified enterobactin (^#^P>0.05/6 and <0.1) compared to growth and/or survival of hvKP1 grown ascites plus solvent alone. Data are Mean ± S.E.M.; N = 3–4. Panel C: Ascites (batch 2) was not supplemented (unconditioned) or supplemented with 0.1mM of Fe (NO_3_)3, FeCl_3_, or FeSO_4_ , and 5% hvKP1 generated conditioned ascites. The growth and/or survival of hvKP1 was significantly increased when grown in ascites supplemented with iron-containing compounds (*P<0.05/3). Data are Mean ± S.E.M.; N = 2.

Although the small size and heat stability of the growth and/or survival enhancing molecule or factor was consistent with an AI, it also suggested the possibility that the observed effects were due to an iron acquisition molecule such as a siderophore(s). Siderophores have been increasingly recognized as critical secreted growth factors in iron-limiting environments [36], which includes the human host [37]. To assess this possibility, an approach utilized to demonstrate that siderophores enable the growth of uncultured bacteria was employed [36]. A limited number of molecules are secreted by the laboratory *E. coli* strain BW25113 (wt). These include the siderophore enterobactin [38], AI-2 molecules, and indole, which can act as a signaling molecule [39]. Conditioned medium from BW25113 and isogenic mutant derivatives deficient in the production of enterobactin (BW25113Δ*entB*, BW25113Δ*entC*), AI-2 molecules (BW25113Δ*luxS*), and indole (BW25113Δ*tnaA*) was generated by overnight growth of these strains in human ascites (batch 1) (heated to 56°C for 30 minutes since BW25113 and derivatives are killed by complement-mediated bactericidal activity present in ascites). Initial titration experiments established that ascites containing a final concentration of 50% conditioned ascites generated by BW25113 resulted in the optimal growth of hvKP1 at a starting inoculum of approximately 10^3^ cfu/ml (data not shown). Growth of hvKP1 in ascites plus conditioned ascites generated from BW25113, BW25113Δ*luxS* and BW25113Δ*tnaA* was similar to that when conditioned ascites generated from hvKP1 (5% final concentration, positive control) was used (Figure 8A). By contrast, growth of hvKP1 in ascites plus conditioned ascites generated from BW25113Δ*entB* and BW25113Δ*entC* was significantly decreased compared to when hvKP1 was grown in ascites supplemented with conditioned ascites generated by the *E. coli* strains sufficient in enterobactin production or with conditioned ascites generated by hvKP1 (Figure 8A). Essentially no growth of hvKP1 was observed in ascites supplemented with a final concentration of 50% of unconditioned ascites heated to 56°C for 30 minutes, which served as a control for the addition of conditioned ascites diluting complement-mediated bactericidal activity in ascites. Therefore, these data demonstrate that the siderophore enterobactin, but not AI-2 molecules or indole, can serve as a growth and/or survival-enhancing factor for hvKP1 in human ascites.

To confirm that enterobactin can serve as a growth and/or survival enhancing factor for hvKP1 in human ascites, hvKP1 was grown in unconditioned human ascites (batch 1) at a starting concentration of approximately 3–4×10^3^ cfu/ml to which solvent alone (0.86% dimethyl sulfoxide (DMSO)) or 40nM, 80nM, 160nM, 320nM, 640nM, and 1.28 µM of purified enterobactin (Sigma Life Sciences (Cat. No. 3910)) were added. The presence of 160nM, 320nM, 640nM, and 1.28 µM of purified enterobactin significantly enhanced the growth of hvKP1 compared to the solvent control (Figure 8B). These concentrations are below the physiologic concentration of enterobactin (8 µM) secreted by an extraintestinal pathogenic strain of *E. coli* [40].

Lastly, since the function of a siderophore is to acquire iron for growth, the addition of iron added to unconditioned human ascites would be hypothesized to enhance growth and/or survival of hvKP1 in this medium. This hypothesis was substantiated when the addition of 0.1mM of Fe (NO_3_)3, FeCl_3_, or FeSO_4_ to unconditioned ascites (batch 2) significantly increased the growth of hvKP1 compared to when these iron containing compounds were not added (Figure 8C). Taken together these data unequivocally demonstrate that the molecule or factor present in conditioned ascites or minimal medium that enhances the growth and/or survival of hvKP1 enables iron acquisition.

### Compared to “classic” *K. pneumoniae* strains, hvKP1 appears to secrete either quantitatively more or biologically more active mediators in ascites that enhance the growth and/or survival of hvKP1

**Figure 9:**
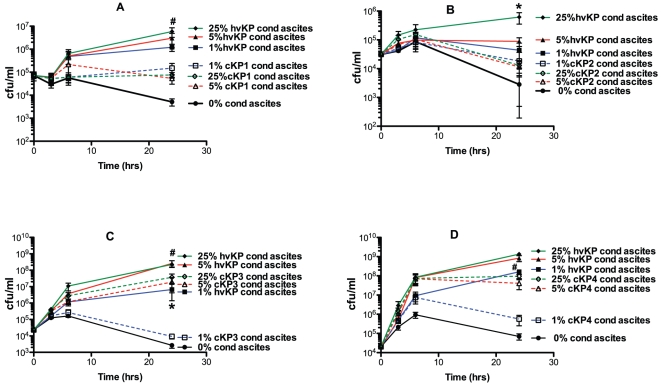
hvKP1 generated conditioned ascites is a more potent enhancer of growth/survival in ascites of cKP1-4 than cKP1-4 generated conditioned ascites. The growth of cKP1-4 was assessed at 0, 3, 6, and 24 hours in human ascites (batch 2) that was heated at 56°C for 30 minutes (Δ56°C) and supplemented with 0%, 1%, 5%, or 25% of hvKP1 or cKP1-4 generated conditioned Δ56°C ascites. Panel A: cKP1; there was a trend for increased growth and/or survival of cKP1 when ascites was supplemented with 25% hvKP1-generated conditioned ascites compared to 25% cKP1-generated conditioned ascites (#P>0.05/3 but <0.05). Panel B: cKP2; the growth and/or survival of cKP2 was significantly increased when ascites was supplemented with 25% hvKP1-generated conditioned ascites compared to 25% cKP2-generated conditioned ascites (*P<0.05/3). Panel C: cKP3; the growth and/or survival of cKP3 was significantly increased when ascites was supplemented with 1% hvKP3-generated conditioned ascites compared to 1% cKP3-generated conditioned ascites (*P<0.05/3) and there was a trend for increased growth and/or survival of cKP1 when ascites was supplemented with 5% and 25% hvKP1-generated conditioned ascites compared to 5% and 25% cKP1-generated conditioned ascites (#P>0.05/3 but <0.05). Panel D: cKP4; there was a trend for increased growth and/or survival of cKP4 when ascites was supplemented with 1% hvKP1-generated conditioned ascites compared to 1% cKP4-generated conditioned ascites (#P>0.05/3 but<0.05). Data are Mean ± S.E.M.; N = 4.

The data presented above (Figure 8B) demonstrated that the siderophore enterobactin was able to enhance the growth and/or survival of hvKP1 in ascites. Whether hvKP1 produces enterobactin is presently unknown, however, hvKP1 and cKP strains undoubtedly produces a number of iron-acquisition molecules that likely function in a similar fashion. Therefore, these data generated the hypothesis that hvKP1 produces either quantitatively more or biologically more active mediators of iron acquisition in ascites that enhance its growth and/or survival in that medium compared to cKP strains. To test this hypothesis hvKP1-generated and “classic” *K. pneumoniae* (cKP1-4)-generated conditioned ascites was made in Δ56°C -ascites (batch 2). Δ56°C -ascites was used due to the variable serum sensitivity of the strains (data not shown). The final titers of the strains used to generate the conditioned medium were similar (1.9×10^9^, 2.5×10^9^, 2.1×10^9^, 7.4×10^8^, and 2.7×10^9^ cfu/ml for hvKP1, CKP1-4 respectively); establishing that potential differences in the concentration of mediators was not due to variability in bacterial load when the conditioned media was processed. The effect of supplementing Δ56°C -ascites (batch 2) with 0%, 1%, 5%, or 25% of hvKP1 or cKP1-4 generated conditioned ascites on the growth and/or survival of the four “classical” *K. pneumoniae* strains was assessed. Supplementation of ascites with hvKP1 generated conditioned ascites significantly increased the growth and/or survival of cKP1-4 compared to when the ascites was supplemented with the homologous conditioned ascites (Figure 9). This finding was consistently observed despite the possibility that cKP strains may not possess receptors for all of the iron-acquisition molecules secreted by hvKP1. As expected, hvKP1-generated conditioned ascites significantly enhanced the growth of itself compared to when cKP-generated conditioned medium was used (data not shown). Taken together, these data demonstrate that hvKP1 either produces quantitatively more of this mediator(s) in ascites that enhances its growth and/or survival in that medium or the molecule(s)/factor(s) produced by hvKP1 are biologically more active (increased affinity for iron or more resistant to host factors) than those produced by cKP strains.

### Compared to “classic” *K. pneumoniae* strains, hvKP1 secretes quantitatively more siderophores that also appear to be biologically more active in ascites

To directly confirm that hvKP1 and cKP strains produce siderophores and to assess whether hvKP1 produces more siderophores than the cKP strains, a quantitative siderophore assay was performed on the hvKP1 and cKP1-4 generated Δ56°C -conditioned ascites used for the experiments described above (Figure 9). The siderophore concentration of hvKP1 was 3 to 7 fold higher than cKP strains and these differences were significantly different (P<0.05/4) (Table 1). These data, in combination with the growth data presented above (Figure 9) enabled us to determine if the effect of hvKP1 conditioned ascites was solely due to having an increased concentration of siderophores or if the siderophores produced had an increased affinity for iron or were more resistant to host factors designed to inactivate siderophores. Examination of Figure 9 demonstrates that supplementation of ascites with 5% hvKP1 conditioned ascites resulted in higher growth of cKP1-4 compared to when ascites was supplemented with 25% cKP1-4 conditioned ascites. Based on quantitative siderophore concentration data (Table 1), the calculated siderophore concentrations in 25% cKP1-4 conditioned ascites were 4.469 µg/mL, 1.950 µg/mL, 3.221 µg/mL, and 2.754 µg/mL respectively. The calculated siderophore concentration in 5% hvKP1 conditioned ascites was 2.738 µg/mL. Therefore, in 3 of 4 instances (cKP1, cKP3, cKP4) 5% hvKP1 conditioned ascites promoted greater bacterial growth despite less or similar amounts of siderophores being present when compared to 25% cKP1-4 conditioned ascites (Table 1). These data strongly support the concept that in addition to producing quantitatively more siderophores, hvKP1 produces biologically more active siderophores. The nature of increased activity is unknown, but one can postulate that siderophores produced by hvKP1 possess an increased stability against host defense mechanisms or heightened efficiency in iron-acquisition in host environment.

**Table 1:** Siderophore concentration in hvKP1 and cKP1-4 generated conditioned ascites.

	Measured Mean ± SEM (µg/mL)	Calculated concentration in 25% conditioned ascites for cKP1-4 and 5% for hvKP1
hvKP1	54.75±0.1166 *	2.738
cKP1	17.87±1.891	4.469
cKP2	7.801±1.514	1.950
cKP3	12.89±0.8523	3.221
cKP4	11.02±0.9765	2.754

*P<0.05/4 , hvKP1 compared to cKP1-4.

## Discussion

Our understanding of the pathogenesis of hvKP, the responsible virulence factors, and the means by which its virulence is enhanced compared to cKP has just begun. Clearly the genotype and phenotype of hvKP has evolved, however the nature of this change is poorly understood. cKP strains appear to be genomically distinct from hvKP [41], [42], despite sharing some common virulence factors with hvKP strains. The presence of a hypermucoviscous phenotype and selected capsular serotypes (primarily serotypes K1 or K2), were initially proposed to be defining characteristics of hvKP [2], [7], [43]. The hypermucoviscous phenotype is an important virulence factor for *K. pneumoniae* [44], regardless of the capsule serotype [45]. The majority, but not all of the strains that cause CA-PLA possess the gene that confers this phenotype, *rmpA* [41], [43], [45], [46]. The biochemical nature of the hypermucoviscous phenotype is unclear but appears to be distinct from the constitutive capsular serotype (e.g. K1, K2) and the surface polysaccharide colanic acid [44]. In some strains, *rmpA* is present on a large virulence plasmid and may be a surrogate marker for other virulence factors [44], [47]. However, recent genomic characterizations established that the genomic background, rather than the hypermucoviscous phenotype defines hvKP pathogenicity [41], [42], [48]. The capsule, especially serotypes K1 and K2, has long been known to be an important virulence factor in cKP [49], [50], [51], [52], [53]. Because the majority of hvKP strains that caused CA-PLA were either a K1 or K2 serotype, these capsular serotypes were initially believed to be the critical virulence factors in this infectious syndrome [7], [43]. However, 23–33% of strains that cause CA-PLA are non-K1/K2 isolates [43], [45], [54]. Further, since the K1 and K2 serotypes were also present in non-CA-PLA clonal groups that were significantly less virulent in a mouse model, the authors concluded that the pathogenic potential of CA-PLA isolates resides with their genomic background, not the capsular serotype [41]. These data do not exclude capsule as an important virulence factor in infection due to hvKP. However, taken together these data support the concept that genes that encode for non-capsular virulence factors are important in the hypervirulent phenotype of hvKP.

In this report a new mechanism that enhances virulence is described. A molecule(s)/factor secreted by hvKP1 significantly enhances its growth and/or survival in human ascites and serum ex vivo and in an mouse infection model that measures metastatic spread after subcutaneous challenge. Although features such as a size of <3kD, heat stability, and growth characteristics in ascites suggested this molecule(s) was a quorum-sensing compound, it proved to be a molecule(s)/factor(s) is involved in iron uptake and is likely a siderophore(s) or another unique iron-acquisition molecule. Although it is known that iron acquisition is critical for virulence, a novel aspect of this observation is that hvKP1 produces quantitatively more siderophores that appear to be biologically more active (increased affinity for iron or more resistant to host factors) than those produced by cKP strains. We are unaware of any published data that considers the virulence potential of a pathogen is quantitatively linked to its iron-acquisition capacity or whether specific iron-acquisition systems are biologically more or less active in Klebsiella. Although increased production of iron acquisition factors could be due to a mutation in the negative regulator Fur, this is unlikely. Supplementation of ascites with hvKP1 generated conditioned LB medium (which is iron replete and therefore Fur repression is active) does not increase its growth and/or survival (Figure 3). This finding is consistent with the normal expression of iron-regulated genes in hvKP1. If in future studies the increased virulence of hvKP can be attributed to specific iron-acquisition molecules, then these compounds would represent potential therapeutic targets or targets for active or passive immunization.

Data presented demonstrates that the addition the siderophore enterobactin to human ascites can enhance the growth and/or survival of hvKP1 in that medium (Figure 8B). But the identity of the molecules(s)/factors(s) secreted specifically by hvKP1 and which serve the same purpose remain undefined. Limited data exists on which factors are present in hvKP versus cKP strains. A bioinformatics analysis of NTUH-K2044, the only sequenced hvKP strain with a completed genome, demonstrated that it possessed on the integrative element ICE*Kp1* orthologues for the siderophore yersinibactin and the siderophore receptor IroN that were not present on the genome of the cKP MGH78578 [42]. In another report, gene clusters for the yersinia high pathogenicity island, *iucABCDiutA*, *iroNDCB*, and *hmu*RSTUV, which encode for iron acquisition systems were identified as being more prevalent in hvKP than cKP isolates[55]. The Kfu system has also been shown to be more prevalent in hvKP strains compared to cKP isolates [56]. Ongoing studies are focusing on the nature and quantity of the mediators present in hvKP1-generated conditioned ascites.

Unlike quorum sensing systems in which AI can self-amplify their production, iron-acquisition systems are iron-regulated and do not autoinduce. Since the concentration of the iron-acquisition mediators produced by hvKP1/cell in the fixed volume of our assay should be the same at low and high titers, it was curious that growth and/or survival only occurred at high titer (Figure 1). One theory would be that ascites contains a host molecule that is capable of binding and inactivating the bacterial iron-acquisition molecules. The host protein lipocalin 2 possesses these properties [57]. Lipocalin 2 would be predicted to be present in ascites. If so, one could hypothesize that when hvKP1 is present in ascites at lower titers the concentration of lipocalin 2 may be sufficient to bind an adequate amount of iron-acquisition mediators to inhibit growth. However by contrast, when hvKP1 is present at a high titer then the concentration of iron-acquisition mediators may exceed the binding capacity of lipocalin 2. This may also be another mechanism that might explain the effect of inoculum on the development of infection. However, once infection is established, one would predict that hvKP is now capable of acquiring iron in a more efficient fashion, which in turn will enhance its ability to grow and/or survive host defenses. This feature may be an explanation for its propensity for metastatic spread from the original site of infection. Further, under conditions in which iron is more available such as hemolysis, tissue necrosis, or a local anaerobic environment, the ability to grow and the overall virulence of hvKP would be predicted to increase and therefore initial infection could be established with a lower bacterial inoculum. These hypotheses are presently being tested.

In summary, the data presented delineates a new mechanism by which hvKP increases its pathogenic potential compared to cKP strains. It is mediated by the ability of hvKP1 to produce quantitatively more and biologically more active iron-acquisition molecule(s)/factor(s) than those produced by cKP strains. It should be noted that this mechanism possesses features of quorum sensing with which it can be confused. This paradigm may be broadly applicable to other extraintestinal gram-negative bacilli.

## Materials and Methods

### Ethics statement

The mouse subcutaneous (SQ) infection model studies was reviewed and approved by the Western New York Veterans Administration Institutional Animal Care Committee. This study was carried out in strict accordance with the recommendations in the Guide for the Care and Use of Laboratory Animals of the National Institutes of Health and all efforts were made to minimize suffering. The procedures for obtaining human serum and ascites were reviewed and approved by the Western New York Veterans Administration Institutional Review Board. Informed consent was used to obtain human blood for the preparation of serum (approval ID # 00063). The Western New York Veterans Administration Institutional Review Board for the process of obtaining ascites waived informed consent (approval ID # 00098). An expedited review was performed because the ascites was collected from de-identified patients who were undergoing therapeutic paracentesis for symptoms due to abdominal distension.

### Strain description

hvKP1 was isolated from the blood and liver abscess in a previously healthy 24 yo male from Buffalo, New York, USA with CA-PLA and metastatic spread to the spleen [6]. It possesses a K2 serotype and is hypermucoviscous. A *luxS* homolog (*ygaG*) was identified in hvKP1 and a LuxS deficient derivative (hvKP1Δ*luxS*) was generated by allelic exchange as described [58]. The *E. coli* laboratory strain BW25113 and isogenic derivatives deficient in the production of enterobactin (BW25113Δ*entB* (CGSC#8704), BW25113Δ*entC* (CGSC#8702)), AI-2 molecules (BW25113Δ*luxS* (CGSC#10100)), and indole (BW25113Δ*tnaA* (CGSC#8309)) were obtained from the Keio Collection knockout library [59].

### Media

hvKP1 was maintained at −80°C in 50% Luria-Bertani (LB) broth and 50% glycerol. Human ascites was collected from individuals were not being treated with antimicrobials and were not infected with human immunodeficiency, hepatitis B and hepatitis C viruses. The ascites was cultured to confirm sterility, divided into aliquots, and stored at −80°C. Each batch was obtained from a different patient and was designated by the date of removal; batch 1 (12/2007) and batch 2 (8/2009). For various in vitro growth studies 100% ascites, Luria-Bertani medium and M9 minimal medium was used.

### *In vitro* growth in ascites, LB and M9 minimal medium

Growth in these media were performed as described with aliquots removed for bacterial enumeration at 0, 3, 6 and 24 hours [30].

### Development of conditioned medium

The bacterial strains, from which conditioned medium was being generated, were grown overnight in ascites, LB or M9 medium (supplemented with antibiotics when appropriate), using a starting inoculum of >1×10^7^ cfu/ml. Aliquots of the overnight cultures were removed for bacterial enumeration. The cultures were then centrifuged at 7000×g for 10 minutes at 4°C. The supernatants were initially passed through a 0.45 µm and then subsequently through a 0.2 µm syringe filter (Corning Inc., Part No. 431229, PES membrane) and stored as aliquots at −80°C. For some experiments conditioned medium was size fractionated using centrifugal filter devices with molecular weight cut-offs of 3 and 10kD (Centricon, Millipore Corp., Bedford, MA).

### Serum bactericidal assay

Complement-mediated bactericidal assays were performed at a starting inoculum of approximately 1×10^5^ cfu/ml as described except that aliquots were removed for bacterial enumeration at 0, 3, 6 and 24 hours [6], [29], [30]. Growth in heat inactivated serum served was a positive control that established that decreased survival was due to complement-mediated bactericidal activity.

### Mouse subcutaneous challenge infection model

This model was developed for the *in vivo* evaluation of hvKP since these strains are capable of gaining entry into the human host via this route, making this model clinically relevant. However, even if hvKP enters the human host through another site (e.g. intestinal tract), SQ challenge will enable an evaluation of the extraintestinal phase of the infectious process. Further and importantly, this route of infection will result in bacteria entering the bloodstream at a physiologically appropriate titer, which in turn, will result in an appropriate host inflammatory response. SQ challenge with an appropriate dose of hvKP1 results in systemic infection. Male CD1 mice (18–22 grams) are challenged SQ with hvKP1. A total of six animals were used per group with three animals from each group euthanized at 24 and 48 hours post-bacterial challenge, after which blood, lungs, kidneys, liver, spleen, and eyes are harvested for enumeration of bacterial colony forming units (cfu). First, blood is obtained from the left ventricle for culture. Next, the right atrium is incised and 5ml of 1x PBS is injected through the left ventricle so that the entire blood volume is flushed from the vasculature. Subsequently, organs are harvested. In this manner bacterial counts solely reflect organ-specific cfu.

### Confirmation of the phenotype of hvKP1 ΔluxS

A bioassay, using the reporter strain BB170 (ATCC number BAA-1117; autoinducer-1 (AI-1) sensor-minus, AI-2 sensor-positive, Km^r^, *luxN*::Tn5; AI-1^+^ AI-2^+^), was used as described [60] to confirm that *luxS* (*ygaG*) was successfully disrupted in hvKP1*ΔluxS*. In brief, BB170 was grown in autoinducer bioassay medium [61] and was exposed to sterile conditioned media generated from the overnight growth of the following strains in autoinducer medium: 1- positive control: BB120 (ATCC number BAA-1116; WT, AI-1 sensor-positive, AI-2 sensor 2-positive, AI-1^+^ AI-2^+^, parent of BB170), 2- negative controls: *E.coli* strains DH5α (*luxS*-minus) and JW2662-1 (CGSC#10100, Keio Collection, *luxS*-minus), and 3 - strains to be tested: hvKP1 and hvKP1*ΔluxS*. Light production was measured every 30 minutes, from 0 to 6 hours using a LUMAT luminometer (Berthold GmbH, Germany, LB 9501). The response of BB170 to the conditioned media from the controls was as expected (data not shown). BB170 produced light in response to the conditioned medium from hvKP1, but by contrast, not from hvKP1*ΔluxS* (data not shown). These data validated the phenotype of hvKP1*ΔluxS*.

### Quantitative siderophore assay

The siderophore concentration in conditioned ascites generated by cKP1-4 and hvKP1 used to assess the effect of hvKP1 conditioned medium and the homologous conditioned medium on the growth of each of the respective cKP strains (Figure 9) was determined using the SideroTec assay (Emergen Bio, Maynooth, Ireland). Standards were prepared by serial dilution of the standard with diluent and were 0, 1.3, 3.1, 6.25, 12.5, 25, 50, and 100 µg/mL. In a flat-bottom 96-well plate, 100 µL of each standard and conditioned ascites were added into wells containing the catalyst reagent (diluted 1∶10 in the dye reagent). The reaction was incubated for 10 minutes, then read every 5 minutes at 630 nm until 50 minutes. For quantitative interpretation, a reference curve was calculated as follows: (OD standard/OD zero standard) x 100. A curve was generated using cubic spline analysis in Prism software. The siderophore concentration in each sample was extrapolated from the reference curve. The incubation time that resulted in the highest concentration for a given sample was used for quantitation, which was 10 minutes for conditioned ascites generated by cKP2-4 and 45 minutes for conditioned ascites generated by cKP1 and hvKP1.

### Statistical Analyses

Data are presented as mean ± SEM. P values of 0.05/n (n =  the number of comparisons) are considered statistically significant based on the Bonferroni correction for multiple comparisons, and P values >0.05/n but <0.1 are considered as representing a trend. To normalize *in vitro* and *in vivo* data log_10_ transformed values were utilized, the area under each curve was calculated, and these areas compared using two-tailed unpaired t tests (Prism 4 for MacIntosh, GraphPad Software Inc.).
